# THE IMPACT OF 3 MONTHS OF SUPERVISED EXERCISE ON CHANGE IN FUNCTION: COMPARISONS BY ARTHRITIS STATUS

**DOI:** 10.1093/geroni/igz038.136

**Published:** 2019-11-08

**Authors:** Lauren M Abbate, Katherine Hall, Megan Pearson, Richard Sloane, Kelli D Allen, Wendy M Kohrt, Miriam C Morey

**Affiliations:** 1 Rocky Mountain Regional VA Medical Center, Aurora, Colorado, United States; 2 Durham Veterans Affairs Health Care system, Durham, North Carolina, United States

## Abstract

Physical activity is an established intervention for the management of arthritis. This study evaluated the effect of 3 months of participation in Gerofit on physical function by arthritis status. Participants, 519 Veterans aged ≥ 65 years self-reported either no arthritis (NA) (49%), upper body arthritis (UB) (8.2%), lower body arthritis (LB) (12.7%), or both upper and lower body arthritis (UB&LB) (30.4%) upon enrollment. Physical function measures [10-meter usual gait speed (m/s) (GS), arm curls (AC), chair stands (CS), and 6-minute walk (yards) (SMW)] were assessed at baseline and follow-up. Mean differences between time points were calculated. At baseline, compared to NA, LB and UB&LB had slower GS (1.10 and 1.06 vs 1.13) and shorter SMW distance (468.8 and 448.8 vs 490.7). All groups tended to increase physical function, with greatest improvement among LB (GS=0.27, AC=2.06, CS=2.52, SMW=42.53). Participation in Gerofit is associated with functional gains, regardless of burden of disease.

